# Effect of Fetal Brain Oxygenation and Volume on Brain Maturation and Neurodevelopmental Outcomes in Congenital Heart Disease

**DOI:** 10.1148/radiol.250651

**Published:** 2025-10-07

**Authors:** Elizabeth George, Jing Liu, Megan Martin, Amy Kuang, Aaron Scheffler, Lauren Christopher, Duan Xu, Patrick McQuillen, Shabnam Peyvandi

**Affiliations:** ^1^Department of Radiology and Biomedical Imaging, University of California, San Francisco, San Francisco, Calif; ^2^Department of Bioengineering, Stanford University, Stanford, Calif; ^3^Department of Epidemiology & Biostatistics, University of California, San Francisco, San Francisco, Calif; ^4^Department of Pediatrics, University of California San Francisco Benioff Children's Hospital, 550 16th St, San Francisco, CA 94158

## Abstract

**Background:**

Neurodevelopmental delay is a cause of long-term morbidity in congenital heart disease (CHD). There are limited data on the effects of the fetal period on later neurodevelopment.

**Purpose:**

To assess the effect of impaired fetal brain oxygenation and fetal brain volume on perinatal brain maturation and early neurodevelopmental outcomes.

**Materials and Methods:**

In this secondary analysis of a single-center prospective study (April 2017 to November 2024), fetuses with CHD and normal fetuses underwent third-trimester fetal brain MRI with T2* mapping to assess oxygenation. Those with CHD underwent neonatal MRI, and neurodevelopmental testing at 30 months of age with the Bayley Scales of Infant and Toddler Development. T2* and brain volume were indexed by sex and gestational age to controls, to generate residuals. The associations of residual fetal T2* and residual fetal brain volume with brain volume growth rate from fetal to neonatal MRI, neonatal white matter (WM) apparent diffusion coefficient and fractional anisotropy, and neurodevelopment at 30 months were assessed using univariable and multivariable linear regression models.

**Results:**

The study included 80 individuals (mean gestational age at fetal brain MRI, 34.0 weeks ± 0.9 [SD]; 52 male fetuses; 60 fetuses with CHD, 20 controls). There was no evidence of an association of residual fetal T2* with perinatal brain growth rate (β = 0.06 [95% CI: −0.00, 0.12]; *P* = .06), WM apparent diffusion coefficient (*P* = .11), WM fractional anisotropy (*P* = .21), or 30-month neurodevelopment (motor, *P* = .99; language, *P* = .89; cognition, *P* > .99). In multivariable analysis, single ventricle physiology was associated with worse motor scores than transposition of the great arteries (β = −19.87 [95% CI: −39.61, −0.13]; *P* = .049), while residual fetal brain volume was positively associated with language score (β = 0.79 [95% CI: 0.05, 1.54]; *P* = .04).

**Conclusion:**

Fetal brain oxygenation was not associated with perinatal brain maturation or early neurodevelopmental outcomes, but fetal brain volume and lesion complexity (as represented by lesion type) were associated with improved language and worse motor outcomes, respectively.

© The Authors 2025. Published by the Radiological Society of North America under a CC BY 4.0 license.

*Supplemental material is available for this article.*

See also the editorial by Huisman in this issue.

## Graphical abstract

**Figure:**
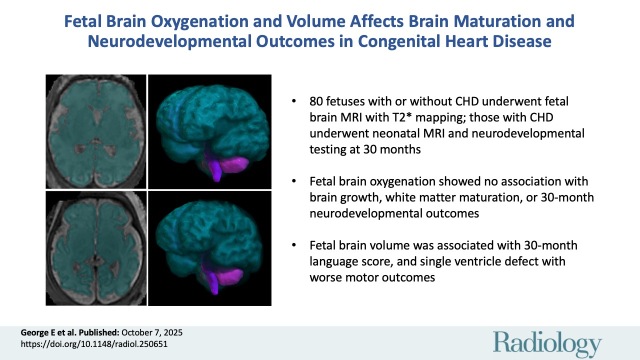

**Summary**

Fetal cerebral oxygenation was not associated with perinatal brain maturation or early neurodevelopmental outcomes, but fetal brain volume and lesion complexity were associated with improved language and worse motor outcomes, respectively.

**Key Results**

■ In this secondary analysis of a prospective study of 80 individuals, individuals with or without congenital heart disease (CHD) underwent fetal brain MRI with T2* mapping to assess fetal brain oxygenation, and those with CHD underwent neonatal MRI and neurodevelopmental testing at 30 months.
■ There was no evidence of an association of fetal brain oxygenation with perinatal brain growth (β = 0.06; *P* = .06), white matter maturation (apparent diffusion coefficient, *P* = .11; fractional anisotropy, *P* = .21), or 30-month neurodevelopmental outcomes (all *P* ≥ .89).
■ Fetal brain volume was positively associated with language score at 30 months (*P* = .04), and single ventricle physiology was associated with worse motor outcomes than transposition of the great arteries (*P* = .049).

## Introduction

Congenital heart disease (CHD) is the most common congenital anomaly, occurring in 6–9 of every 1000 live births (1). Over the past several decades, mortality associated with CHD has decreased (2), and neurodevelopmental delay has emerged as a cause of long-term morbidity (3). Neurodevelopmental delay in CHD is thought to be due to a combination of factors, including altered prenatal and perinatal cardiac physiology, neonatal surgery, genetic or epigenetic factors, and environmental factors (4,5). The fetal and early neonatal period are known to play a key role because even children with dextro-transposition of the great arteries, who undergo definitive corrective surgery as neonates, develop neurodevelopmental delay (6).

There are structural correlates to neurodevelopmental delay in CHD, including brain dysmaturity starting in late fetal life (7), and an increased risk of acquired neonatal brain injury (8). These structural brain changes are likely partly due to alterations in cardiac physiology that result in altered fetal cerebral oxygenation, which can be measured using various MRI techniques, such as T2* mapping, phase-contrast imaging, and blood oxygenation level–dependent functional MRI (9–12). Prior studies have shown lower fetal cerebral oxygenation in fetuses with CHD compared with controls, with minimal differences across lesion types (9,10).

Impaired neonatal preoperative, intraoperative, and postoperative brain oxygenation assessed using near-infrared spectroscopy has shown variable associations with brain volume and neurodevelopmental outcomes in early life (13). There are limited data on the effects of such brain alterations in the fetal period on later brain maturation and neurodevelopmental outcomes. Obtaining such data is critical because the fetal period may represent a time period when neuroprotective interventions can be implemented for long-term benefit. Therefore, the purpose of this study was to assess the effect of impaired fetal brain oxygenation and fetal brain volume on perinatal brain maturation and early neurodevelopmental outcomes in a cohort of individuals with CHD.

## Materials and Methods

### Study Sample

The study protocol for this secondary analysis of a single-center prospective longitudinal study was approved by the institutional review board for human research at the University of California, San Francisco. Informed consent was obtained from all participants (ie, pregnant individuals), and the study was compliant with the Health Insurance Portability and Accountability Act. This study involved fetuses with a prenatal diagnosis of severe CHD between April 2017 and November 2024 who were expected to undergo neonatal cardiac surgery at the University of California, San Francisco (Fig 1). In addition, healthy pregnant individuals with no fetal anomalies and normal fetal US and echocardiographic examinations were enrolled from the low-risk obstetric clinic at the university between June 2017 and December 2019 as a control group (9,14). Pregnant individuals with prenatally diagnosed genetic or extracardiac fetal abnormalities, twin gestation, intrauterine growth restriction, significant uteroplacental disease (such as preeclampsia), or significant maternal disease (diabetes or hypertension requiring medication use) were excluded.

**Figure 1:**
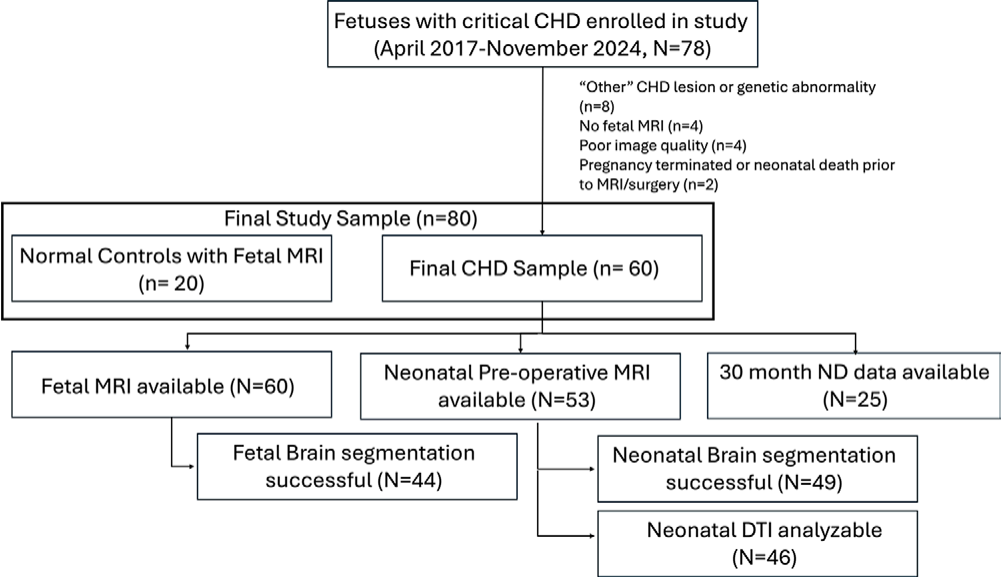
Flowchart of the study sample. CHD = congenital heart disease, DTI = diffusion tensor imaging, ND = neurodevelopmental.

Detailed clinical data were collected for all participants and infants in the CHD group, including demographic characteristics, cardiac lesion type, perinatal course (including gestational age at birth), and length of hospital stay. In addition, all participants were administered a questionnaire at enrollment that included information on maternal education (ordinal scale from less than seventh grade to graduate degree). The CHD cohort mainly included fetuses with either dextro-transposition of the great arteries or single ventricle physiology. Transposition of the great arteries was defined as great vessel malposition with the aorta arising from the right ventricle and the pulmonary artery arising from the left ventricle, with or without a ventricular septal defect. Single ventricle physiology was defined as having only one functioning ventricle, requiring palliative surgical intervention for survival in the newborn period.

### MRI Procedures

All participants in the CHD and control groups underwent fetal brain MRI in the third trimester, without sedation, using a 3-T scanner (Discovery MR750; GE Medical Systems) with a 32-channel cardiac coil. Sequences included single-shot fast spin-echo T2-weighted imaging and a four-dimensional multiecho gradient-echo acquisition for T2* mapping using a previously published protocol (9). Single-shot fast spin-echo parameters were as follows: echo time, 100 milliseconds; repetition time, 4 seconds; section thickness, 3 mm; matrix, 256 × 192; and field of view, 28–32 cm. The four-dimensional multiecho gradient-echo acquisition had the following parameters: field of view, 32.0 × 32.0 cm; section thickness, 6 mm; matrix, 160 × 160 × 30; repetition time, 24.0–24.2 milliseconds; eight echo times of 2.0–22.0 milliseconds, with 2.9-millisecond echo spacing; flip angle, 20°; readout bandwidth, ±125 kHz; and scan time, 140 seconds.

The continuously acquired data were reconstructed into a three-dimensional image at 10 time frames (14 seconds per frame) using a multicoil compressed sensing method (k-t SPARSE-SENSE [15,16]) with an acceleration factor of approximately 8. Processing involved an institutional pipeline with semiautomatic segmentation, and the average signal intensity of the whole fetal brain at each echo time was obtained to derive the mean fetal brain T2* (Fig 2) (9). Single-shot fast spin-echo T2-weighted imaging in multiple planes and slice-to-volume reconstruction were used to create a three-dimensional volume, from which fetal brain volume was derived using an automated pipeline (17–19).

**Figure 2:**
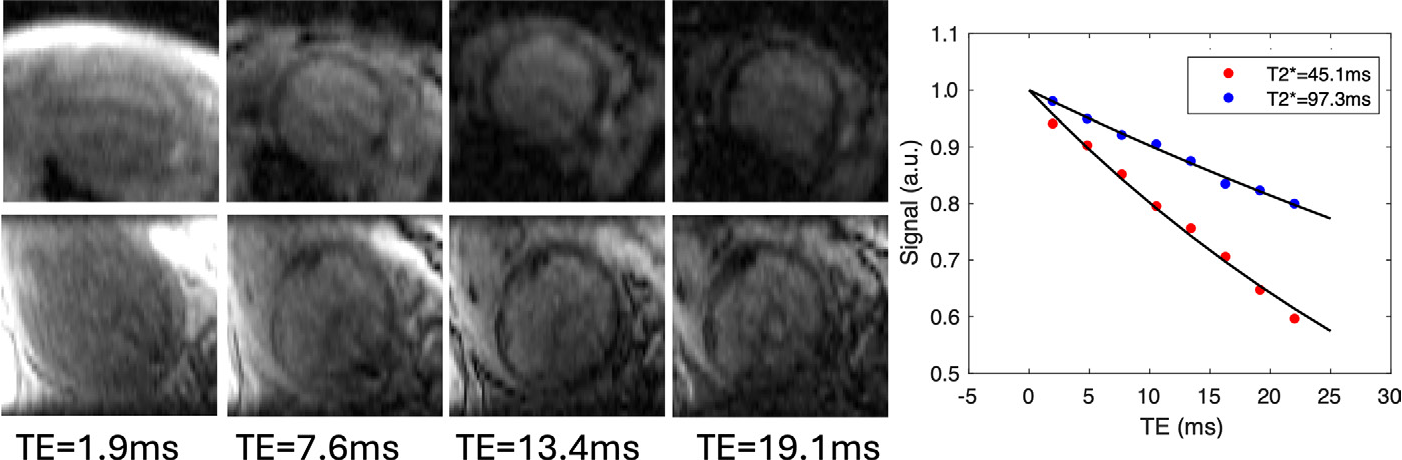
Calculation of T2* relaxation time at fetal brain MRI. Images in the top row are in a male fetus with single ventricle physiology at 33.9 weeks of gestation, with a T2* of 45.1 milliseconds. Images in the bottom row are in a male fetus with transposition of the great arteries at 34.1 weeks of gestation, with a T2* of 97.3 milliseconds. Four axial gradient-echo MRI scans at echo time (TE) 1.9–19.1 milliseconds are depicted. The graph on the right depicts the T2* decay curves in the two fetuses, with signal intensity in arbitrary units (a.u.) plotted on the y-axis and echo time plotted on the x-axis (red corresponds to the top row of images; blue corresponds to the bottom row of images).

Neonates underwent preoperative brain MRI as soon as they could be safely transported to the MRI scanner, typically within the first week of life. Postoperative brain MRI was performed after neonatal cardiac surgery and before discharge. MRI examinations were performed on a 3-T Discovery MR750 system (GE HealthCare) and included the following sequences: three-dimensional isotropic T1-weighted inversion-recovery spoiled gradient-recalled echo (repetition time msec/echo time msec/inversion time msec, 8.7/3.5/450; section thickness, 1 mm); T2-weighted fast spin-echo (repetition time msec/echo time msec, 5000/120; section thickness, 3 mm; in-plane resolution, 2 × 2 mm); three-dimensional isotropic T2-weighted (3000/93; section thickness, 1 mm); diffusion tensor imaging (5000/63; section thickness, 1.8 mm; no gap; in-plane resolution, 2 × 2 mm; 30 directions; b value, 700 sec/mm^2^); and susceptibility-weighted imaging (20,21).

### Imaging Evaluation

Preoperative and postoperative MRI scans were reviewed by two authors (E.G. and P.M., with 5 and >20 years of experience, respectively) in unblinded consensus reads for white matter (WM) injury (22,23), which was classified as none, minimal (1–3 foci, all <2 mm), moderate (>3 foci or any foci >2 mm), or severe (>5% of WM volume) (24). The cumulative WM injury classification from the pre- and postoperative brain MRI examinations was included as an independent variable in the analyses of neurodevelopmental outcomes. Preoperative brain volume was calculated from T1- and T2-weighted images using the publicly available processing pipeline from the Developing Human Connectome Project (25,26). The rate of growth of the brain was calculated as the change in brain volume from fetal MRI to neonatal preoperative MRI divided by the time (in weeks) between the two MRI examinations. Diffusion images from neonatal preoperative MRI were processed using Diffusion Toolbox (FMRIB Software Library), and the apparent diffusion coefficient and fractional anisotropy of the WM were masked using the segmentations on T2-weighted images from the Developing Human Connectome Project pipeline (25).

### Neurodevelopmental Assessment

All participants in the CHD group were invited back for neurodevelopmental testing of their child at 30 months of age with the Bayley Scales of Infant and Toddler Development (BSID) (motor, language, and cognition domains), administered by a licensed psychometrician. During the time period of this study, there was a version update of the BSID, such that one child was evaluated using BSID III, while the remaining children were evaluated using BSID IV. Given the systematic difference in scores between versions, the child evaluated with BSID III was excluded from the analysis; thus, numerical BSID IV motor, language, and cognition scores were used as the outcomes.

### Statistical Analysis

A previous report provides a detailed cross-sectional comparison between the CHD and control groups (9). In the current study, longitudinal data collected in the CHD cohort are reported. The data from the control group were only used to create standardized metrics for the primary predictor variables of interest. The primary variables of interest were residual fetal T2* and residual fetal brain volume. Fetal T2* and brain volume depend on gestational age; hence, residual values for fetal T2* and brain volume were calculated based on the difference in individual values from the expected value in the control group estimated using a linear regression model adjusted for gestational age and sex. The primary outcomes of interest were rate of growth in brain volume from fetal to neonatal preoperative MRI, neonatal preoperative WM apparent diffusion coefficient and fractional anisotropy, and BSID IV score in each of the three domains of neurodevelopment at 30 months.

Clinical and demographic independent variables included in the univariable analyses were selected a priori based on known association with neurodevelopmental outcomes; these included CHD lesion type (transposition of the great arteries or single ventricle physiology), maternal education, gestational age at birth, length of neonatal hospital stay, and moderate-to-severe cumulative WM injury, when appropriate. CHD lesion type affects cardiovascular physiology as well as neonatal surgical treatment; specifically, patients with transposition of the great arteries undergo corrective surgery, while those with single ventricle physiology undergo palliative surgery. Maternal education was categorized as high (college graduate or higher) or low (partial college or specialized degree or lower). Length of neonatal hospital stay was used as a surrogate for complexity of hospital course (3). An independent variable was not included in a given analysis if it chronologically occurred after the dependent variable (eg, length of neonatal hospital stay was not included as an independent variable for perinatal brain growth rate but was included for neurodevelopmental outcomes).

Univariable and multivariable linear regression models were used to estimate associations of predictor variables with BSID score in each neurodevelopmental domain. Variables that were near significant (*P* < .1) in univariable analysis were included in the multivariable analysis. The difference in adjusted *R*^2^ value for the multiple predictor regression models with and without the variable of interest was used to determine the variance explained for each neurodevelopmental domain. *P* < .05 was considered to indicate a statistically significant difference or correlation. All analyses were performed by two authors (E.G. and S.P.) using Stata version 14.0 (StataCorp).

## Results

### Study Sample Characteristics

A total of 80 fetuses were included in this study (mean gestational age at fetal brain MRI, 34.0 weeks ± 0.9 [SD]; gestational age range, 32.0–36.3 weeks; 52 male [65%] and 28 female [35%] fetuses), including 60 fetuses with CHD (mean gestational age, 34.0 weeks ± 0.8; 43 male fetuses) and 20 normal fetuses (mean gestational age, 34.0 weeks ± 1.0; 11 female fetuses). Of the 78 individuals with fetuses with CHD who were enrolled, eight were excluded due to lack of fetal MRI examination (ie, claustrophobia) or poor image quality (ie, T2* could not be calculated). Eight individuals with fetuses with CHD other than transposition of the great arteries or single ventricle physiology (ie, coarctation or tetralogy of Fallot) were enrolled; these individuals were excluded from the analysis due to the small sample size. Two additional participants were excluded due to pregnancy termination or neonatal death before MRI, leading to a final population of 60 maternal-fetal pairs who underwent fetal MRI (Fig 1). In the CHD group, neurodevelopmental assessment at 30 months was completed in 25 children and is pending in 17 children (Table S1). Characteristics of the study sample are provided in Table 1.

**Table 1:** Characteristics of the Study Sample

Characteristic	All Individuals (*N* = 80)	CHD Group (*n* = 60)	Control Group (*n* = 20)	*P* Value*
Gestational age at fetal MRI (wk)	34.0 ± 0.9	34.0 ± 0.8	34.0 ± 1.0	.91
Fetal sex^†^				
Male	52/80 (65)	43/60 (72)	9/20 (45)	.03
Female	28/80 (35)	17/60 (28)	11/20 (55)	
Fetal brain volume (mL)	231.8 ± 27.0	230.6 ± 27.4	236.6 ± 26.2	.52
Fetal brain T2* (msec)^‡^	84.0 (69.7–94.1)	79.6 (66.3–89.8)	95.25 (89.0–104.4)	<.001
Gestational age at birth (wk)	38.9 ± 1.0	38.8 ± 1.0	39.3 ± 1.1	.07
CHD lesion type^†^				
Transposition of great arteries	NA	33/60 (55)	NA	NA
Single ventricle physiology	NA	27/60 (45)	NA	NA
Maternal education^†^^§^^ǁ^			NA	NA
Low	NA	26/56 (46)	NA	NA
High	NA	30/56 (54)	NA	NA
Gestational age at preoperative MRI (wk)^#^	NA	39.4 ± 1.0	NA	NA
Age at preoperative MRI (d)^ǁ^^#^	NA	5.4 ± 3.3	NA	NA
Moderate-to-severe WM injury at preoperative MRI^†^^#^**^††^	NA	12/52 (23)	NA	NA
Gestational age at postoperative MRI (wk)^#^	NA	44.4 ± 4.6	NA	NA
Age at postoperative MRI (d)^#^	NA	40.4 ± 40.0	NA	NA
Cumulative moderate-to-severe WM injury^†^^ǁ^^#^**	NA	18/56 (32)	NA	NA
Length of neonatal hospital stay (d)	NA	53.1 ± 54.1	NA	NA

Note.—Except where indicated, data are means ± SDs. CHD = congenital heart disease, NA = not applicable, WM = white matter.

* *P* value for comparison of CHD group and control group, obtained with the Student *t* test, χ^2^ test, or Wilcoxon rank sum test as appropriate.

^†^ Data are numbers of individuals, with percentages in parentheses.

^‡^ Data are medians, with IQRs in parentheses.

^§^ High maternal education defined as college graduate or higher.

^ǁ^ Data missing in four individuals with CHD.

^#^ Controls did not undergo neonatal MRI.

** Moderate-to-severe WM injury defined per McQuillen et al (24).

^††^ Data missing for eight individuals with CHD.

### Growth Rate of Brain Volume

The mean growth rate of brain volume from fetal MRI to neonatal preoperative brain MRI in the CHD group was 14.5 mL/wk ± 2.7. There was no evidence of an association between residual fetal T2* and growth rate of brain volume between the two time points (β = 0.06 [95% CI: 0.00, 0.12]; *P* = .06) (Fig 3, Table 2).

**Figure 3:**
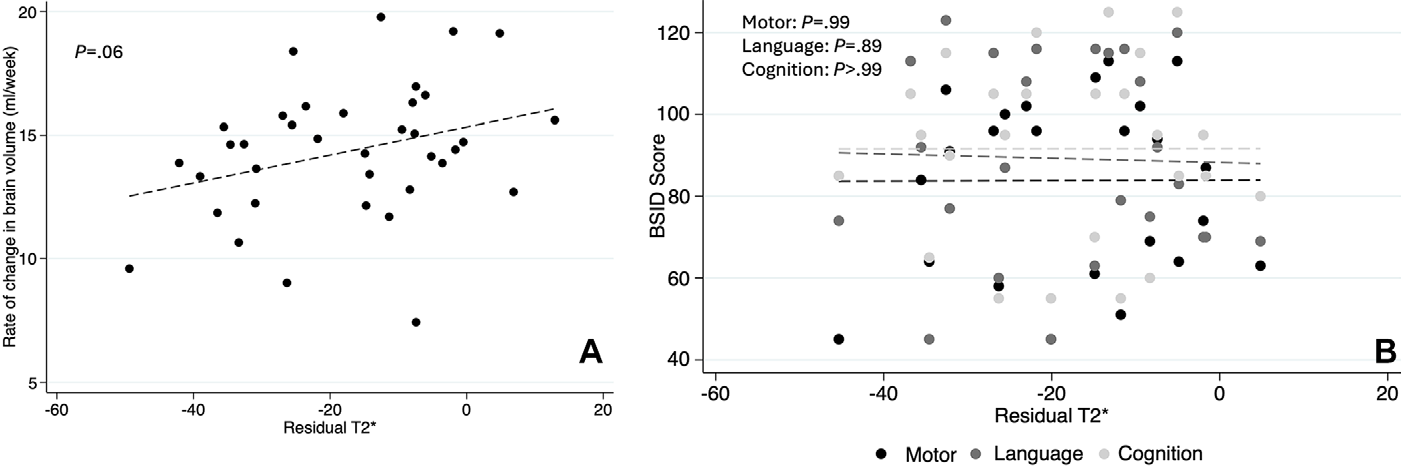
Association of residual T2* at fetal brain MRI with **(A)** rate of change in brain volume and **(B)** 30-month Bayley Scales of Infant and Toddler Development (BSID) scores. **(A)** Scatter plot shows no evidence of an association between residual fetal T2* and the rate of change in brain volume from fetal MRI to neonatal preoperative MRI. Residual T2* is plotted on the x-axis, and rate of change in brain volume is plotted on the y-axis, with the line representing the linear fit. **(B)** Scatter plot shows no evidence of an association between residual fetal T2* and 30-month neurodevelopmental outcomes. Residual T2* is plotted on the x-axis, and BSID motor score (black), language score (dark gray), and cognition score (light gray) are plotted on the y-axis, with the corresponding lines representing the linear fits.

**Table 2:** Univariable Analysis of Predictors of Brain Volume Growth Rate, WM Apparent Diffusion Coefficient, and WM Fractional Anisotropy

Predictor	Brain Volume Growth Rate (*n* = 39)		Apparent Diffusion Coefficient (*n* = 46)		Fractional Anisotropy (*n* = 46)	
	β Coefficient	*P* Value	β Coefficient	*P* Value	β Coefficient	*P* Value
Gestational age at birth	0.91 (−0.11 to 1.92)	.08	NA	NA	NA	NA
Gestational age at neonatal MRI	NA	NA	−0.02 (−0.04 to 0.00)	.01	0.00 (0.00 to 0.01)	.24
High maternal education*	−0.24 (−2.01 to 1.52)^†^	.78	0.00 (−0.03 to 0.03)^‡^	.97	0.00 (0.00 to 0.01)^‡^	.28
Moderate-to-severe preoperative WM injury^§^	1.71 (−0.43 to 3.84)	.11	0.02 (−0.02 to 0.06)	.31	0.00 (−0.01 to 0.01)	.57
Lesion type						
Transposition of great arteries	Reference		Reference		Reference	
Single ventricle physiology	−0.19 (−2.05 to 1.67)	.84	−0.01 (−0.04 to 0.02)	.59	0.00 (0.00 to 0.01)	.45
Residual fetal T2*	0.06 (0.00 to 0.12)^ǁ^	.06	0.00 (0.00 to 0.00)^#^	.11	0.00 (0.00 to 0.00)^#^	.21
Residual fetal brain volume	0.03 (−0.01 to 0.08)	.10	0.00 (0.00 to 0.00)	.98	0.00 (0.00 to 0.00)	.51

Note.—Data in parentheses are 95% CIs. All analyses were linear regression. NA = not applicable, WM = white matter.

* High maternal education defined as college graduate or higher.

^†^ Data missing in four individuals.

^‡^ Data missing in three individuals.

^§^ Moderate-to-severe WM injury defined per McQuillen et al (24).

^ǁ^ Could not be calculated in two individuals.

^#^ Could not be calculated in one individual.

### WM Maturation

Among neonates with CHD, the mean WM apparent diffusion coefficient was 1.32 × 10^−3^ mm^2^/sec ± 0.05 × 10^−3^, and the mean WM fractional anisotropy was 0.16 ± 0.01. Higher gestational age at neonatal MRI was associated with lower WM apparent diffusion coefficient at neonatal MRI (β = −0.02 [95% CI: −0.04, 0.00]; *P* = .01). There was no evidence of an association between residual fetal T2* or residual fetal brain volume and neonatal WM apparent diffusion coefficient (residual fetal T2*: β = 0.00 [95% CI: 0.00, 0.00], *P* = .11; residual fetal brain volume: β = 0.00 [95% CI: 0.00, 0.00], *P* = .98) or fractional anisotropy (residual fetal T2*: β = 0.00 [95% CI: 0.00, 0.00], *P* = .21; residual fetal brain volume: β = 0.00 [95% CI: 0.00, 0.00], *P* = .51) (Table 2).

### Neurodevelopmental Outcomes

At 30 months, the mean BSID IV score for all children with CHD was 83.8 ± 22.4 in the motor domain, 89.2 ± 24.3 in the language domain, and 91.6 ± 21.9 in the cognition domain. The following predictors were associated with lower scores in the motor, language, and cognition domains with *P* < .1 in the univariable analysis: greater length of neonatal hospital stay, lower maternal education, cumulative moderate-to-severe WM injury, and CHD lesion type (Table 3, Fig 4). In addition, lower fetal residual brain volume in the third trimester was associated with lower language score with *P* < .1 (β = 0.57 [95% CI: −0.08, 1.21]; *P* = .08) (Table 3) and was included in the multivariable analysis for this outcome. There was no evidence of an association between residual fetal T2* and 30-month motor (β = 0.01 [95% CI: −0.73, 0.74]; *P* = .99), language (β = −0.05 [95% CI: −0.85, 0.75]; *P* = .89), or cognition (β = 0.00 [95% CI: −0.72, 0.72]; *P* > .99) score in univariable analysis (Table 3).

**Table 3:** Univariable Analysis of Predictors of Motor, Language, and Cognition Scores of the Bayley Scales of Infant and Toddler Development at 30 Months of Age (*n* = 25 Children)

Predictor	Motor		Language		Cognition	
	β Coefficient	*P* Value	β Coefficient	*P* Value	β Coefficient	*P* Value
Gestational age at birth	0.24 (−11.11 to 11.59)	.97	2.89 (−9.37 to 15.15)	.63	0.05 (−11.06 to 11.17)	.99
Length of neonatal hospital stay	−0.34 (−0.63 to −0.05)	.02	−0.42 (−0.72 to −0.12)	.01	−0.35 (−0.63 to −0.07)	.02
High maternal education*	17.61 (−0.36 to 35.57)	.05	25.35 (7.24 to 43.46)	.01	18.78 (1.72 to 35.83)	.03
Cumulative moderate-to-severe WM injury^†^	−17.44 (−37.12 to 2.25)	.08	−22.56 (−43.27 to −1.84)	.03	−21.07 (−39.60 to −2.54)	.03
Lesion type						
Transposition of great arteries	Reference		Reference		Reference	
Single ventricle physiology	−23.6 (−40.0 to −7.19)	.01	−22.27 (−40.91 to −3.62)	.02	−22.33 (−38.61 to −6.06)	.01
Residual fetal T2*	0.01 (−0.73 to 0.74)	.99	−0.05 (−0.85 to 0.75)	.89	0.00 (−0.72 to 0.72)	>.99
Residual fetal brain volume^‡^	0.27 (−0.29 to 0.83)	.32	0.57 (−0.08 to 1.21)	.08	0.45 (−0.14 to 1.03)	.17

Note.—Data in parentheses are 95% CIs. All analyses were linear regression. WM = white matter.

* High maternal education defined as college graduate or higher. Data missing in one individual.

^†^ Moderate-to-severe WM injury defined per McQuillen et al (24).

^‡^ Data missing in eight individuals.

**Figure 4:**
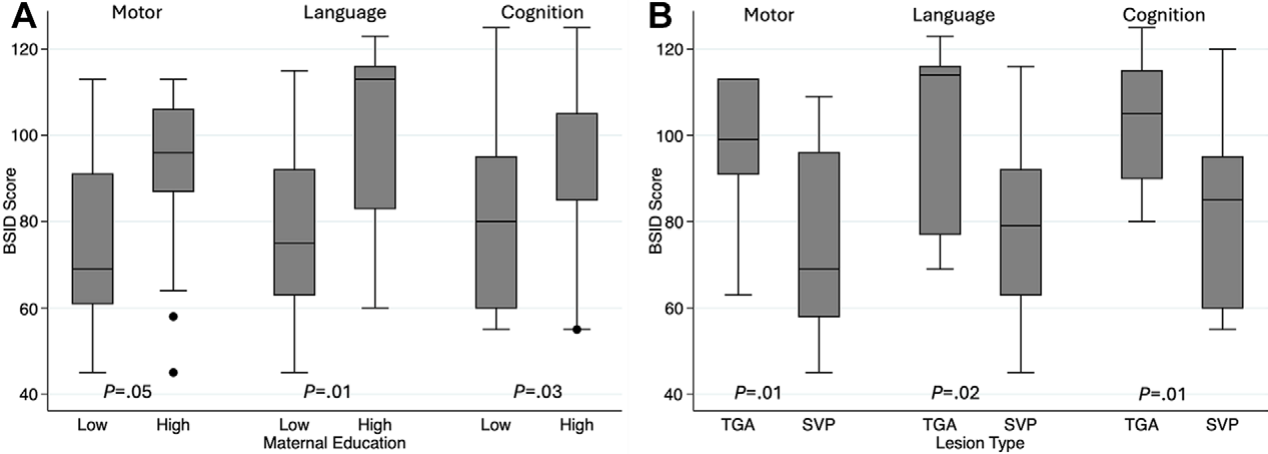
Box plots depict the effect of **(A)** maternal education (low or high) and **(B)** congenital heart disease lesion type (transposition of the great arteries [TGA] or single ventricle physiology [SVP]) on motor, language, and cognition scores of the Bayley Scales of Infant and Toddler Development (BSID) at 30 months of age in univariable analysis. In the box plots, the box indicates the IQR, the center line indicates the median, whiskers indicate 1.5× IQR, and individual dots represent outliers. High maternal education was defined as college graduate or higher; low maternal education was defined as partial college or specialized degree or lower. Single ventricle physiology was associated with worse BSID scores in all domains compared with transposition of the great arteries, and low maternal education was associated with worse language and cognition BSID scores than high maternal education.

Since length of neonatal hospital stay was highly correlated with CHD lesion type (mean length of stay, 36.1 days ± 34.2 for transposition of the great arteries vs 74.9 days ± 66.5 for single ventricle physiology; *P *= .01), only lesion type was included in the multivariable analysis due to its known relevance as a predictor of the outcomes of interest. In multivariable analysis, single ventricle physiology was associated with a 19.87-point lower mean motor score compared with transposition of the great arteries (β = −19.87 [95% CI: −39.61, −0.13]; *P *= .049) (Table 4). High maternal education (college graduate or higher) was associated with a 34.60-point higher mean language score compared with low maternal education (β = 34.60 [95% CI: 2.49, 66.71]; *P *= .04). After accounting for gestational age and sex, every 1-mL increase in fetal brain volume was associated with a 0.79-point increase in language score (β = 0.79 [95% CI: 0.05, 1.54]; *P *= .04) (Table 4). Figure 5 shows images from fetal brain MRI in two fetuses with single ventricle physiology with different exposures and developmental outcomes. There was no evidence of an association of high maternal education (β = 13.80 [95% CI: −3.04, 30.64]; *P *= .10), cumulative moderate-to-severe WM injury (β = −7.01 [95% CI: −27.88, 13.85]; *P *= .49), or lesion type (β = −15.19 [95% CI: −34.04, 3.67]; *P *= .11) with cognition score at 30 months (Table 4).

**Table 4:** Multivariable Analysis of Predictors of Motor, Language, and Cognition Scores of the Bayley Scales of Infant and Toddler Development at 30 Months of Age

Predictor	Motor		Language		Cognition	
	β Coefficient	*P* Value	β Coefficient	*P* Value	β Coefficient	*P* Value
High maternal education*	13.48 (−4.15 to 31.11)	.13	34.60 (2.49 to 66.71)	.04	13.80 (−3.04 to 30.64)	.10
Cumulative moderate-to-severe WM injury^†^	−1.27 (−23.12 to 20.58)	.91	13.94 (−27.82 to 55.69)	.48	−7.01 (−27.88 to 13.85)	.49
Lesion type						
Transposition of great arteries	Reference		Reference		Reference	
Single ventricle physiology	−19.87 (−39.61 to −0.13)	.049	−3.57 (−29.39 to 22.26)	.77	−15.19 (−34.04 to 3.67)	.11
Residual fetal brain volume^‡^	NA	NA	0.79 (0.05 to 1.54)	.04	NA	NA

Note.—Data in parentheses are 95% CIs. All analyses were linear regression. NA = not applicable, WM = white matter.

* High maternal education defined as college graduate or higher. Data missing in one individual.

^†^ Moderate-to-severe WM injury defined per McQuillen et al (24).

^‡^ Data missing in eight individuals.

**Figure 5:**
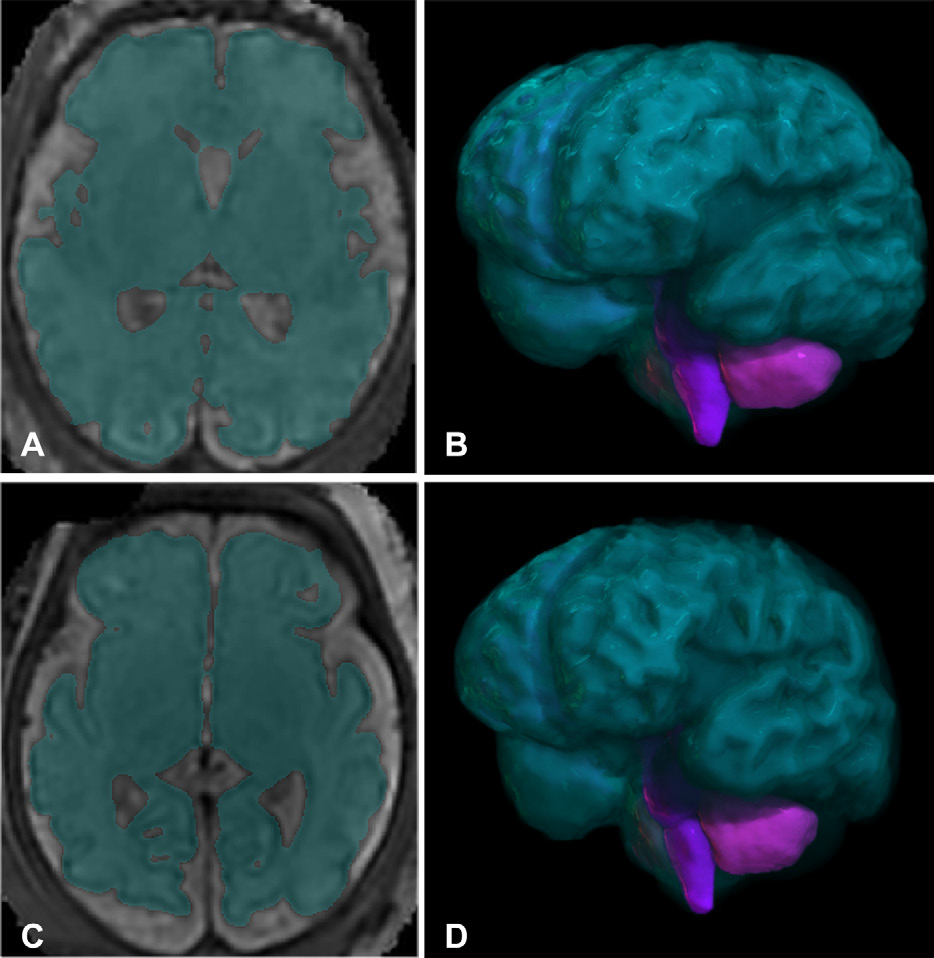
Images from fetal brain MRI in two fetuses with single ventricle physiology. **(A)** Axial T2-weighted slice-to-volume reconstructed image with overlaid brain volume segmentation and **(B)** three-dimensional volume rendering of the brain in a 34-week male fetus with a brain volume of 275 mL (residual brain volume, 9.5 mL), high maternal education, and 30-month Bayley Scales of Infant and Toddler Development (BSID) language score of 116. High maternal education was defined as college graduate or higher. **(C)** Axial T2-weighted slice-to-volume reconstructed image with overlaid segmentation and **(D)** three-dimensional volume rendering of the brain in a 34-week female fetus with a brain volume of 200 mL (residual brain volume, −60.8 mL), low maternal education, and 30-month BSID language score of 45. Low maternal education was defined as partial college or specialized degree or lower. Supratentorial brain is shown in teal, and infratentorial brain is shown in purple.

For the motor score, the CHD lesion type explained 12% of the variance, and the multivariable model explained 25% of the variance. For the language score, maternal education explained 20% of the variance, and residual fetal brain volume explained 14% of the variance; the multivariable model explained 46% of the variance.

## Discussion

There are limited data on the direct effect of abnormal fetal brain oxygenation, as assessed via T2* mapping, and fetal brain development, as assessed via brain volume, on neonatal brain maturation and early childhood neurodevelopmental outcomes in congenital heart disease. In this secondary analysis of a prospective longitudinal study, we begin to disentangle the variable contributions of prenatal factors to neurodevelopmental outcomes. Whole-brain fetal T2* in late gestation was not found to be associated with brain maturation or neurodevelopmental outcomes. Larger fetal brain volume was associated with higher language scores at 30 months (*P* = .04), and including fetal brain volume in the multivariable model greatly increased the variance explained. This finding is in line with a previously published single-center study (27) that demonstrated that fetal brain volume was an important factor influencing neurodevelopmental outcomes in young children. Similar to other studies, we also demonstrated that cardiac lesion complexity (as represented by cardiac lesion type;* P* = .049) and social determinants of health (as represented by maternal education;* P* = .04) are important factors for motor and language outcomes, respectively, in this population.

We and others have shown that fetal brain T2*, a measure of cerebral oxygenation, is lower in fetuses with CHD compared with controls (9,10). Chronic hypoxia has been shown in animal models to impair proliferation, neurogenesis, and migration in the subventricular zone, and thus is an important biomarker of brain health in CHD (28). In this longitudinal study, fetal brain T2* in late gestation but was not associated with brain growth. In animal models, oxygen tension has been shown to be critical for axon integrity and myelination, through the oligodendrocyte precursor cell hypoxia inducible factor signaling pathway (29). There are no prior clinical studies, to our knowledge, on the effect of oxygenation on WM maturation in CHD. While the CHD cohort in our study demonstrated gestational age–related WM maturation, fetal brain oxygenation was not associated with global WM maturation.

Fetal cerebral T2* provides a composite measure of brain oxygenation (ie, accounting for both delivery and consumption) and may reflect compensation in baseline cerebral metabolism and adaptation to an environment of low oxygen and nutrient delivery. Thus, this fetal brain MRI biomarker appears to reflect the known aberrations in fetal cardiovascular physiologic processes in CHD and their impact on the brain. However, brain oxygenation may vary in response to a variety of fetal and maternal factors, and T2* was measured at a single time point in our study. In our study, fetal brain T2* was not associated with early neurodevelopmental outcomes. Fetal oxygenation is one of the early brain alterations in CHD, but there are additional perinatal and postnatal factors, including CHD lesion complexity and hospital course, that may be more proximally causal for neurodevelopmental outcomes. In prior literature, pre-, peri-, and postoperative brain oxygenation assessed using various metrics derived from near-infrared spectroscopy have been variably associated with neurodevelopmental outcomes among those with CHD undergoing neonatal surgery (13,30–34). Social determinants of health are likely to contribute at multiple points to later outcomes, the causal pathways of which are unclear and possibly interrelated. In addition, while we assessed global cerebral oxygenation, regional variability in oxygenation may be more directly relevant to specific functional outcomes.

Various optical and MRI-based techniques exist to estimate brain oxygenation in children (35), some of which have been applied to fetuses with CHD (9–12). While optical techniques have limited penetration, MRI-based techniques are not limited by depth and allow quantification of both vascular (arterial and venous) (11) and parenchymal oxygen content (9,10,12). T2* mapping is one such technique to determine the relative deoxyhemoglobin content of tissues, but it does not disentangle various aspects of cerebral oxygenation, including oxygen delivery and consumption, and is also dependent on cerebral blood flow effects. Quantitative susceptibility mapping, with its ability to differentiate paramagnetic from diamagnetic effects, offers advantages over T2* mapping and can be used to assess cerebral oxygen metabolism. Thus, the lack of association between T2* and outcomes in our study may reflect inherent technique limitations; studies using other tools such as quantitative susceptibility mapping are warranted.

There are limited prior data on the effect of fetal brain volume on outcomes in infants and young children. Importantly, in our study fetal brain volume was strongly associated with neurodevelopmental outcomes at 30 months, particularly language scores after adjusting for other known predictors (ie, lesion complexity and maternal education). This is consistent with a single-center study that found an association between fetal brain volume and neurodevelopmental outcomes at 2 years of age in CHD (36). Our findings on the short-term effects of fetal brain volume on neurodevelopmental outcomes emphasize the importance of the fetal environment as a critical period for implementation of neuroprotective measures to improve outcomes in CHD.

Neurodevelopmental outcome prediction in CHD patients is complex, with various clinical, genetic, and environmental influences, both before and after birth. Similar to prior reports, our findings demonstrate the importance of lesion complexity and sociodemographic factors (22,27). Social determinants of health have been shown to be associated with various aspects of CHD, including incidence, fetal diagnosis, mortality, and postsurgical outcomes, and thus influence neurodevelopmental outcomes at many stages (37). In a study of toddlers with single ventricle physiology, maternal education was independently associated with 30-month motor outcomes (22). Furthermore, a stimulating home environment has been shown to affect 22-month cognitive and 5-year executive function outcomes more than CHD type and brain injury severity do (38,39). Various mechanisms, beyond the direct maternal-infant relationship, may contribute to the neurodevelopmental effects of social determinants of health, including systemic inequities, access to quality care, social and economic factors, physical environment, and provider bias (40,41).

Our study has several limitations. While this study has a relatively large sample size for prospectively collected fetal and neonatal brain MRI and early childhood neurodevelopmental outcomes, the findings may not be generalizable given its single-center nature. Thus, larger, multicenter studies are needed to confirm our findings. Second, 27% of fetal MRI examinations and 6% of neonatal MRI examinations could not be processed through the automated pipelines we used for this study; this is in line with previous success rates using these pipelines. Third, we used apparent diffusion coefficient and fractional anisotropy as metrics of global WM maturation. These do not fully capture regional variations in WM myelination and maturation, which could be assessed using tract-specific diffusion tensor imaging analyses in future studies. Finally, in this study, we focused on 30-month neurodevelopmental outcomes, and prior studies have shown that outcomes at this age are only modestly associated with later outcomes (42); long-term follow-up is necessary.

In severe congenital heart disease (CHD) requiring neonatal surgery, fetal life is a critical period for brain development. In this study, fetal cerebral oxygenation was not associated with perinatal growth rate of brain volume, white matter maturation, or early neurodevelopmental outcomes, but fetal brain volume and lesion complexity were associated with improved language and worse motor outcomes, respectively. Our findings highlight the importance of the fetal period as a target for future neuroprotective trials. Future neuroprotective clinical trials must include interventions in the fetal period to optimize brain growth in utero, and thereby decrease vulnerability to subsequent postnatal risk factors.
